# Trehalose: a potent enhancer of antioxidant responses in *Brassica juncea* L. under arsenic toxicity

**DOI:** 10.1080/15592324.2026.2706952

**Published:** 2026-07-23

**Authors:** Ambika Choudhary, Monika Choudhary, Neetika Kimta, Nidhi Bhardwaj, Sunil Puri, Mehidi Kassim Ahmed, Tabarak Malik

**Affiliations:** a School of Biological and Environmental Sciences, Shoolini University of Biotechnology and Management Sciences, Solan, India; b Centre for Advanced Innovation Technologies, VSB-Technical University of Ostrava, Ostrava-Poruba, Czech Republic; c Department of Chemistry, Faculty of Science, University of Hradec Kralove, Hradec Kralove, Czech Republic; d A.M. Dogliotti College of Health Sciences, University of Liberia, Monrovia, Liberia; e Department of Biomedical Sciences, Institute of Health, Jimma University, Ethiopia; f Division of Research and Development, Lovely Professional University, Phagwara, Punjab, India; g Department of Microbiology, Graphic Era (Deemed to be University), Dehradun, Uttarakhand, India

**Keywords:** Trehalose, arsenic, oxidative stress, *Brassica juncea*, antioxidant defense, osmoprotection, phytoremediation

## Abstract

Arsenic contamination poses a major threat to crop productivity by impairing the photosynthetic machinery and inducing oxidative stress. This study explored the protective role of exogenous trehalose (25, 50, and 75 mM) in mitigating arsenic-induced physiological and biochemical disturbances in *Brassica juncea* at 30, 60, and 90 d after sowing. As stress significantly reduced photosynthetic pigments, including chlorophyll a (up to 72.91%), chlorophyll b (77.71%), total chlorophyll (56.49%), carotenoids (33%–43%), xanthophylls (42%–43%), and anthocyanins (38.82%). Concurrently, oxidative stress markers increased markedly, with malondialdehyde rising by 22%–46% and hydrogen peroxide (H₂O₂) by 12%–59%, indicating severe ROS-induced cellular damage. Foliar application of trehalose effectively alleviated these negative effects in a dose-dependent manner. Trehalose (75 mM) showed the highest efficacy, enhancing chlorophyll a by 49%–88%, chlorophyll b by 56%–78%, total chlorophyll by 52%–66%, carotenoids by 42%–48%, and xanthophylls by 44%–58% compared to As-stressed plants. It also substantially improved the anthocyanin content and decreased MDA levels by 25%–29%, reflecting reduced lipid peroxidation and improved antioxidant defense. While H₂O₂ levels displayed some variability, trehalose consistently moderated its accumulation under As stress. Overall, trehalose significantly restored the pigment profiles, reduced oxidative damage, and enhanced stress tolerance, with 75 mM emerging as the most effective concentration. These results highlight trehalose as a promising and eco-friendly strategy to improve plant resilience and productivity under arsenic-contaminated conditions.

## Graphical abstract

**Figure:**
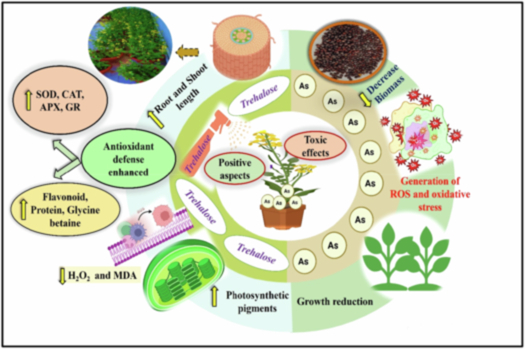

## Introduction

The 20th most common element in the environment, arsenic (As), has a concentration of 1.5–3 mg/kg in the Earth's crust.^1^ ^,^ ^2^ More than 200 arsenic-bearing minerals have been identified in nature, and the most prevalent are sulfide which include realgar, arsenopyrite, and orpiment.^3^ Both natural and anthropogenic activities have significantly increased arsenic contamination, particularly in agricultural soils.^4^ Plants can easily absorb arsenic and store it in their edible parts, which means that arsenic can enter the food chain. It presents severe threats to the health of plants, animals, and humans.^5^

On a cellular level, the effect of arsenic on plants is that it binds to the sulfhydryl groups of enzymes and thus disrupts essential metabolism pathways. Arsenic is also known to inhibit physiological processes such as transpiration, stomatal conductance as well as photosynthesis.^6^ ^,^ ^7^ One of the main impacts of arsenic toxicity on plants is the high production of reactive oxygen species (ROS). High levels of ROS lead to lipid peroxidation, membrane instability, protein oxidation, and damage to DNA.^7^

Plants, in turn, respond by turning on an endogenous antioxidant defense system that includes enzymatic (superoxide dismutase, catalase and ascorbate peroxidase (SOD, CAT, APX)) and non-enzymatic (proline and glutathione) components to scavenge ROS and alleviate oxidative stress.^8^ These antioxidant systems help maintain the cellular redox balance and membrane stability under stress conditions. Under extreme stress conditions, endogenous antioxidants are often inadequate for preventing oxidative stress. Hence, the exogenous application of protective molecules such as nitric oxide can improve the antioxidant status of plants, which, in turn, may decrease the oxidative stress caused by As.^9^

Trehalose (Tre), a non-reducing disaccharide, plays an important role in plant abiotic stress tolerance through osmotic adjustment, membrane stabilization, and ROS detoxification. The regulation of trehalose concentrations within the cells is necessary for the survival of the plant under stress factors such as heat, drought, heavy metals, and pesticides.^10^ It progresses plant growth and strengthens stress tolerance.^11^ Recent research indicates that the exogenous application of Tre under Cd toxicity in *Oryza sativa* L. enhancing the action of antioxidant defense system and increases the growth of plants and also reduces the toxic impact of Cd.^12^ For example,^13^ studied that the external application of trehalose positively impacted the growth and physiology of maize seedlings as well as their antioxidant defense mechanisms under Cr stress.

Heavy metal uptake and detoxification in plants have been well studied, but the mechanisms of tolerance and hyperaccumulation remain unclear. Therefore, interest in metal transport systems in plants has increased.^14^ ^,^ ^15^ The mechanisms of metal tolerance and accumulation can only be understood with a thorough knowledge of metal classification, compartmentalization, and distribution in various organs and cell structures of plants.^16^ ^,^ ^17^ Against this backdrop, the Brassicaceae family, particularly *Brassica juncea* L., has gained attention for its high biomass and remarkable heavy metal accumulation capacity. It is an efficient phytoextractor with high metal accumulation and translocation capacity.^18^ Despite these beneficial characteristics, arsenic exposure induces severe structural and physiological damage in *B. juncea*, leading to impaired photosynthesis and disruption of the antioxidant defense system. Therefore, developing strategies to enhance stress tolerance and detoxification capacity in mustard plants is essential.^19^ Therefore, understanding the role of trehalose in enhancing arsenic tolerance, antioxidant defense, and membrane stability in *B. juncea* is of considerable importance.

The novelty of the current study lies in its comprehensive investigation of the ameliorative role of exogenous trehalose against arsenic-induced toxicity in *B. juncea* under different developmental stages (30-, 60-, and 90-d). The study also evaluated the concentration-dependent effects of trehalose (25, 50, and 75 mM) to identify the most effective dose for alleviating arsenic toxicity. Moreover, various studies have been conducted on the Osmo protectant like proline in heavy metal stress,^20^ but no study has investigated the ameliorative effect of trehalose on arsenic stress in *B. juncea* plants. In addition, the present study provides an integrated assessment of photosynthetic pigments, oxidative stress markers, and enzymatic antioxidant defence systems under arsenic toxicity. Furthermore, the study demonstrates that trehalose effectively alleviates oxidative damage by improving chlorophyll stability, carotenoid and xanthophyll content, and membrane integrity. This study highlights the efficiency of trehalose as a strong Osmo protectant capable of improving physiological and morphological characteristics as a consequence of heavy metal stress in an agriculturally important oilseed crop with phytoremediation potential. In conclusion, the current research presents new results on the Tre concentration-dependent and developmental function to facilitate stress tolerance and provides an environmentally friendly solution to enhance crop resistance and food safety in As-contaminated areas.

## Materials and methods

### Chemicals and instrument used

The chemicals utilized for the treatments are Na_2_HAsO_4_·7H_2_O [Sodium arsenate (Himedia)] and C_12_H_22_O_11_·2H_2_O Tre dihydrate [Tre dihydrate (Himedia)], and the instruments used for the treatments were centrifuge (T-24BL), spectrophotometer (Thermo Fisher scientific-3020-563).

### Experimental design

**Figure 1.**
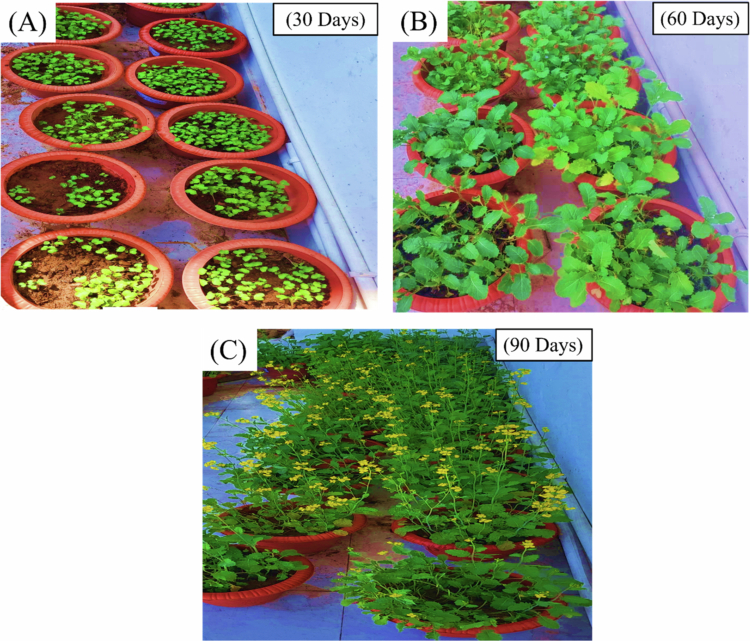
Morphological development of *B. juncea* at different growth stages: (A) 30 d old plants (initial growth stage); (B) 60 d old plants (vegetative stage); (C) 90 d old plants (reproductive stage).

A pot experiment was carried out at the Agricultural Farm at Shoolini University (Figure 1). Model pot trials were conducted in pot measuring 18 × 16 cm. The pots were filled with a mixture of soil and organic manure in a 4:1 ratio. *B. juncea* seeds were surface sterilized using 0.01% HgCl₂ for 1 min and rinsed three to four times with sterile distilled water.

The experimental treatments included arsenic (As 0.4 mM), trehalose (25, 50, and 75 mM), and combined treatments (As + Tre 25, As + Tre 50, and As + Tre 75 mM). Distilled water served as the control.

Following sowing, the plants were cultivated under natural conditions and harvested at 30, 60, and 90 d after sowing. At each stage, different parameters like photosynthetic, ROS associated stress indicators, and enzymatic and as well as non-enzymatic activities.

### Photosynthetic parameters

#### Chlorophyll content

Arnon,^21^ method was followed for the evaluation of chlorophyll contents. Fresh plant material (0.5 g) was thoroughly crushed in chilled 80% (v/v) acetone (4 mL) using a pre-cooled pestle and mortar and subsequently centrifuged at 13,000 rpm for 20 min at 4 °C. The optical density of the supernatant was recorded at 645 and 663 nm using a UV-visible spectrophotometer. Chlorophyll a, and chlorophyll b and total chlorophyll, contents were calculated and reported as mg g^−1^ fresh weight (FW) using the following equations:

$$\mathrm{Total chlorophyll}\;\;=\;\frac{[(A64\;\times \;20.2)\;+\;(A663\;\times \;8.02)]\;}{1000\;\times \;w}\;\times \;v,$$

$$\mathrm{Chlorophyll a}=\frac{[(A663\;\times \;12.7)\;-\;(A645\;\times \;2.69)]\;}{1000\;\times \;w}\times \;v,$$

$$\mathrm{Chlorophyll b}=\frac{[(A645\;\times \;22.9)\;-\;(A663\;\times \;4.89)]\;}{1000\;\times \;w}\;\times \;v.$$

where w = weight of plant sample; v = volume of plant extract.

#### Total carotenoid content

The method of Maclachlan and Zalik^22^ was employed to estimate the total carotenoid concentration. For this purpose, 0.5 g of fresh plant tissue was homogenized in 4 mL of chilled 80% acetone using a pre cooled pestle and mortar and centrifuged for 20 min at 13,000 rpm at 4 °C. The supernatant was collected for the evaluation of total carotenoid content by taking the absorbance at 480 and 510 nm. The total carotenoid concentration was calculated and expressed as mg g^−1^ FW using the following equation:

$$\mathrm{Carotenoid}\;\mathrm{content}\;=\;\frac{[(A480\;\times \;7.6)\;-\;(A510\;\times \;1.49)]\;}{1000\;\times \;w}\;\times \;v,$$

where w = weight of plant sample; v = volume of plant extract.

#### Total xanthophyll content

The method described by Lawrence,^23^ was used for the determination of the xanthophyll level. Dried samples (50 mg) were treated with 30 mL of a multi-solvent extractant (hexane: acetone: absolute alcohol: toluene; 10:7:6:7 v/v) in 100 mL conical flasks. The mixture was then agitated for 10–15 min to ensure thorough extraction. Subsequently, 2 mL of 40% methanolic potassium hydro-oxide was added, and the flask was refluxed at 56 °C for 20 min. The sample was then placed for 1 h, after which 30 mL of hexane was added. Shaking of the flask was done for 1 min, followed by the addition of 10% sodium sulfate solution to make a final volume up to100 mL. The flask was again kept in the dark for 1 h after shaking the flask for 1 min. The upper phase was transferred to a 50 mL volumetric flask and brought to volume with hexane, and the absorbance was recorded at 474 nm. The total xanthophyll content was expressed as mg/g FW using the following equation:

$$\mathrm{Xanthophyll content}\;=\;\frac{A474\times D}{w\times 236},\;$$

where D = final dilution; w = weight of sample taken; and 236 = specific-absorptivity (trans-lutein in gl^−1^).

#### Anthocyanin content

The method of Mancinelli,^24^ was followed for the estimation of anthocyanin content. Fresh plant tissue (0.5 g) was homogenized in 3 mL of extraction mixture consisting of methanol, distilled water and HCl at a ratio of 79:20:1 (v/v/v). The homogenate was then centrifuged at 13,000 rpm, and the supernatant was collected. The absorbance of the extract was recorded at 530 and 657 nm, and the total anthocyanin content was expressed as mg g^−1^ FW using the following equation:

A=A530−(0.25×A657),

$$\mathrm{Xanthophyll}\;\mathrm{content}=\frac{A\times \mathrm{MW}}{ɛ}\times 1000.$$

where ɛ = molar absorptivity (cyanidin-3-glucoside, 26900).

MW = molecular weight of cyanidin-3-glucoside (449.2).

### Oxidative stress markers

#### Malondialdehyde content

The malondialdehyde (MDA) content was determined according to the method of Singh and Upadhyay.^25^ Fresh seedlings (1 g) were homogenized in 0.1% (w/v) trichloroacetic acid (TCA), and the homogenate was centrifuged to obtain a clear supernatant. Subsequently, 20% (w/v) thiobarbituric acid (TBA) was added to the supernatant. The reaction mixture was then incubated at 95 °C and rapidly cooled to room temperature. The absorbance of the resulting solutions was recorded at 532 and 600 nm. The MDA content was expressed as μ mol g^−1^ FW in the given equation:

$$\mathrm{MDA}=\frac{\mathrm{Abs}\times \mathrm{total}\;\mathrm{volume}\times 1000}{\mathrm{Extinction}\;\mathrm{coefficient}\times \mathrm{sample}\;\mathrm{volume}\times \mathrm{weight}\;\mathrm{of}\;\mathrm{plant}\;\mathrm{tissue}},$$

where, extinction coefficient = 155 mM^−1^ cm^−1^.

#### Hydrogen peroxide content

The hydrogen peroxide (H_2_O_2_) content was calculated using the method of Velikova et al.^26^ Fresh plant tissue (100 mg) was homogenized in 0.1% (w/v) TCA, and the homogenate was centrifuged to obtain a clear supernatant. Then, 0.5 mL of the supernatant was mixed with 0.4 mL of potassium phosphate buffer (PPB) and 0.8 mL of potassium iodide solution. The absorbance of the reaction mixture was recorded at 390 nm. H_2_O_2_ served as the reference standard, and the H_2_O_2_ content was expressed as μ mol g^−1^ FW.

### Antioxidant defense system

#### Enzymatic antioxidants

For the estimation of antioxidative enzymes, including superoxide dismutase (SOD), catalase (CAT), ascorbate peroxidase (APX), and glutathione reductase (GR). Fresh plant tissue (1 g) was homogenized in a mortar and pestle with 3 mL of sodium carbonate buffer (50 mM, pH 10.2). The homogenate was centrifuged at 5000 rpm for 20 min, and the supernatant was used for analysis of the activities of the antioxidative enzymes.

##### Catalase.

Catalase (CAT) was determined following the method of.^27^ The reaction mixture consisted of 50 μL of plant extract, 2.650 mL of 100 mM of PPB (pH 7.0) and 300 μL of H_2_O_2_ (150 mM). The decrease in absorbance was recorded at 240 nm, and the CAT activity was calculated using the equation given below:

$$\mathrm{CAT}\;\mathrm{activity}=\frac{\mathrm{Change}\;\mathrm{in}\;\mathrm{Abs}\;\min^{-1}\times \mathrm{total}\;\mathrm{volume}\;(\mathrm{mL})}{\mathrm{Extinction}\;\mathrm{coefficient}\;\mathrm{sample}\;\mathrm{volume}\;(\mathrm{mL})\times \mathrm{weight}\;\mathrm{of}\;\mathrm{tissue}\;(g)},$$

where, extinction coefficient = 43.6 M^−1^ cm^−1^

$$\mathrm{Specific}\;\mathrm{unit}\;\mathrm{activity}\;(\mathrm{mol}\;U\;mg^{-1}\mathrm{FW})=\frac{\mathrm{Unit}\;\mathrm{activity}\;{(\mathrm{Unit}\;\min}^{-1}g^{-1}\mathrm{FW})}{\mathrm{Protein}\;\mathrm{content}\;(\mathrm{mg}\;g^{-1}\mathrm{FW})}.$$

FW = fresh weight of the tissue.

##### Ascorbate peroxidase.

The activity of ascorbate peroxidase (APX) was estimated according to the protocol described by Nakano and Asada.^28^ The reaction mixture consisted 50 μL of enzyme extract, 3 mL each of ascorbate (5 mM) and H_2_O_2_ (0.5 mM), and 2.370 mL of phosphate buffer (100 mM, pH 7.0). The decrease in absorbance was recorded at 290 nm, and the APX activity was calculated using the equation below:

$$\mathrm{APX}\;\mathrm{activity}=\frac{\mathrm{Change}\;\mathrm{in}\;\mathrm{Abs}\;\min^{-1}\times \mathrm{total}\;\mathrm{volume}\;(\mathrm{mL})}{\mathrm{Extinction}\;\mathrm{coefficient}\;\mathrm{sample}\;\mathrm{volume}\;(\mathrm{mL})\times \mathrm{weight}\;\mathrm{of}\;\mathrm{tissue}\;(g)},\;$$

where, extinction coefficient = 2.8 mM^−1^ cm^−1^

$$\mathrm{Specific}\;\mathrm{unit}\;\mathrm{activity}\;(\mathrm{mol}\;U\;mg^{-1}\mathrm{FW})=\frac{\mathrm{Unit}\;\mathrm{activity}\;{(\mathrm{Unit}\;\min}^{-1}g^{-1}\mathrm{FW})}{\mathrm{Protein}\;\mathrm{content}\;(\mathrm{mg}\;g^{-1}\mathrm{FW})},$$

FW = fresh weight of the tissue.

##### Superoxide dismutase.

Standard methodology of^29^ was followed for the determination of superoxide dismutase (SOD) activity. In the reaction mixture, 300 μL of nitroblue tetrazolium (NBT; 96 μM), 300 μL Triton X-100 (0.6%), and 1700 μL of Na_2_CO_3_ buffer (50 mM, pH 10) were added to the test cuvettes. The reaction was initiated by the addition of 300 μL of hydroxylamine hydrochloride (20 mM, pH 6.0) and 300 μL of ethylene diamine tetra acetic acid (EDTA; 0.1 mM). After 2 min, 100 μL of enzyme extract was added to the reaction mixture. The absorbance was recorded at 540 nm. The percent inhibition of NBT reduction were calculated below in the given equation:

$$x=\frac{\mathrm{change}\;\mathrm{in}\;\mathrm{Abs}\;\min^{-1}\left(\mathrm{blank}\right)-\mathrm{change}\;\mathrm{in}\;\mathrm{Abs}\;\min^{-1}\left(\mathrm{sample}\right)}{\mathrm{change}\;\mathrm{in}\;\mathrm{Abs}\;\min^{-1}\left(\mathrm{blank}\right)}\times 100,$$

where *x* is the % inhibition caused by 100 μL of the sample.

50% inhibition is caused by $=\frac{50\times 100}{x}$ = *y* μL of the sample.

##### Glutathione reductase.

Glutathione reductase (GR) activity was estimated according to the method described by Carlberg and Mannervik.^30^ The reaction mixture contained 2 mL of PPB (50 mM, pH 7.0), 300 µl of EDTA (3 mM), NADPH (0.1 mM), and oxidized glutathione (1 mM), and 100 µl of enzyme extract. The decrease in absorbance was recorded at 340 nm, and the activity of GR was calculated by given equation:

$$\mathrm{GR}\;\mathrm{activity}=\frac{\mathrm{Change}\;\mathrm{in}\;\mathrm{Abs}\;\min^{-1}\times \mathrm{total}\;\mathrm{volume}\;(\mathrm{mL})}{\mathrm{Extinction}\;\mathrm{coefficient}\;\mathrm{sample}\;\mathrm{volume}\;(\mathrm{mL})\times \mathrm{weight}\;\mathrm{of}\;\mathrm{tissue}\;(g)},\;$$

where, extinction coefficient = 6.22 mM^−1^ cm^−1^

$$\mathrm{Specific}\;\mathrm{unit}\;\mathrm{activity}\;(\mathrm{mol}\;\mathrm{U m}g^{-1}\mathrm{FW})=\frac{\mathrm{Unit}\;\mathrm{activity}\;{(\mathrm{Unit}\;\min}^{-1}\;g^{-1}\mathrm{FW})}{\mathrm{Protein}\;\mathrm{content}\;(\mathrm{mg}\;g^{-1}\mathrm{FW})},\;$$

FW = fresh weight of the tissue.

#### Non-enzymatic antioxidants

##### Flavonoid content.

Flavonoid levels were quantified following the methodology of Agbo et al.^31^ Fresh plant tissue (500 mg) was homogenized in 3 mL of methanol, and the homogenate was centrifuged to obtain clear supernatant. To the supernatant, 4 mL of double distilled water (DDW), 0.3 mL of sodium nitrite (NaNO_2_), and 0.3 mL of aluminum chloride (AlCl_3_) were added. The reaction mixture was allowed to stand, after which 2 mL of sodium hydroxide (NaOH) and 2.4 mL of DDW were added, resulting in the development of a pink-colored complex. The absorbance of the reaction mixture was recorded at 510 nm. Rutin was used as the reference standard for calibration. The flavonoid content was expressed as mg/g FW.

##### Protein content.

The protein content of the leaf samples was determined using the method of Lowry et al.^32^ Initially, 2 g of leaf material were accurately weighed and homogenized in 10–20 mL of phosphate buffer using a pestle and mortar. The homogenate was centrifuged at 7500 rpm for five minutes, and the resulting supernatant was collected for analysis. Standard protein solutions with concentrations of 0, 0.2, 0.4, 0.6, 0.8, and 1 mL were prepared in separate test tubes and mixed with the sample extracts. The total volume in each tube was adjusted to 1 mL, with a blank containing 0 mL of extract serving as a control. To each tube, including the blank, 5 mL of alkaline copper reagent was added, and mixed thoroughly. Under alkaline conditions, copper ions reacted with peptide bonds to form a complex, which was allowed to stand for 10 min. Subsequently, 0.5 mL of Folin–Ciocalteu reagent was added to each tube and mixed well. After 30 min of incubation in the dark at room temperature, the reduction of the Folin–Ciocalteu reagent by the copper–peptide complex produced a blue coloration. The intensity of this color was measured spectrophotometrically at 660 nm to determine the protein concentration.

##### Glycine betaine content.

The Grieve et al.^33^ method was used for the estimation of the GB content. Dried seedlings (1 g) were homogenized to obtain a clear solution. After filtration, 1 mL of the supernatant was combined with 0.2 mL of potassium triiodide solution and 1 mL of 2 M HCl. The mixture was cooled and agitated thoroughly, followed by the addition of 2.0 mL of ice-cooled distilled water and 20 mL of 1–2 dichloromethane, respectively. The absorbance of the separated organic phase was measured at 365 nm. Quantification was carried out using a calibration curve prepared with known concentrations of GB. The GB content was expressed as μ mol g^−1^ FW.

### Statistical analysis

Statistically analysis was conducted using Prism version 5.02 software. The experimental data are shown as the mean ± standard deviation (SD) of three trials. Significant difference among treatments were calculated using one way analysis of variance (ANOVA) followed by Bonferroni’s multiple comparison test (*p* < 0.05).

The experimental data are shown as the mean ± standard deviation (SD) of three trials.

## Results and discussion

### Photosynthetic parameters

#### Chlorophyll content

##### Chlorophyll content (30 d).

**Figure 2.**
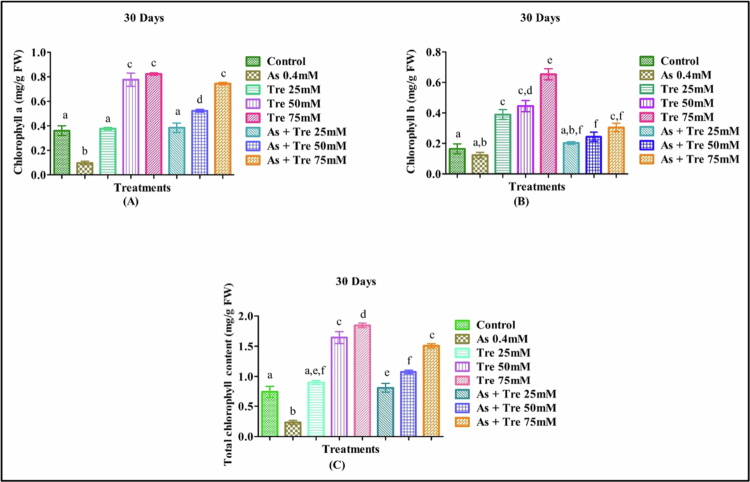
Effect of As and Tre on 30 d old plants of *B. juncea* under various concentrations in comparison with the control plant (A) chlorophyll a, (B) chlorophyll b, and (C) total chlorophyll content. Experimental data represents the mean of triplicates ± standard deviation (SD). Statistical analysis was performed using a one-way ANOVA, with a Bonferroni post-hoc test employed to identify significant differences (*p* < 0.05). Within the figures, distinct superscripts indicate treatments that are significantly different from one another. Treatments included control (without arsenic), and a gradient of arsenic concentration (0.4 mM) and different Tre concentrations (25, 50, and 75 mM).

Arsenic stress significantly decreased the chlorophyll a, b, and total chlorophyll content by 72.98%, 25.15%, and 68.60% in 30 d old *B. juncea* plants. Among all the treatments combined treatment of As + Tre 75 mM significantly restored the chlorophyll content by 56.28%, 59.63%, and 84.60% compared with As-treated plants alone, indicating the protective role of trehalose in maintaining photosynthetic pigment stability under As-stress conditions (Figure 2).

##### Chlorophyll content (60 d).

**Figure 3.**
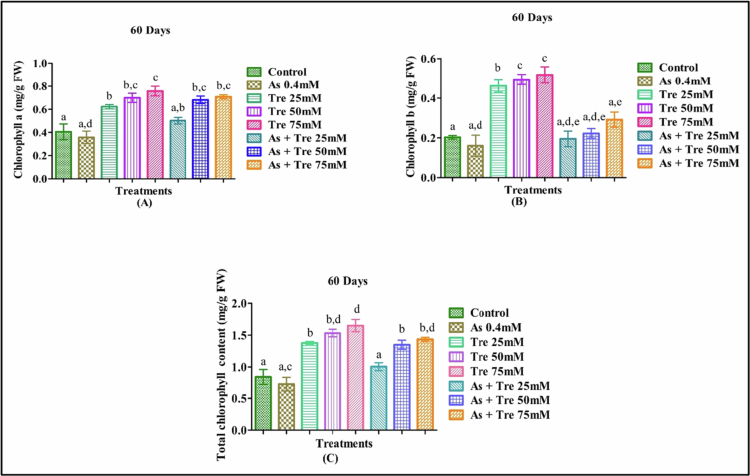
Effect of As and Tre on 60 d old plants of *B. juncea* under various concentrations in comparison with the control plant (A) chlorophyll a, (B) chlorophyll b, and (C) total chlorophyll content. Experimental data represents the mean of triplicates ± standard deviation (SD). Statistical analysis was performed using a one-way ANOVA, with a Bonferroni post-hoc test employed to identify significant differences (*p* < 0.05). Within the figures, distinct superscripts indicate treatments that are significantly different from one another. Treatments included a control (without arsenic), and a gradient of arsenic concentration (0.4 mM) and different Tre concentrations (25, 50, and 75 mM).

In 60-d-old *B. juncea* plants, arsenic stress caused a significant decline in the chlorophyll a, chlorophyll b, and total chlorophyll content by 11.90%, 35.08%, and 29.17%, respectively. However, the accumulation of trehalose reversed the negative effects of As in a concentration-dependent manner. The greatest improvement was observed in the As + Tre 75 mM treatment, which increased of 49.53%, 56.11%, and 52.11% in chlorophyll a, chlorophyll b, and total chlorophyll compared with As treated plants (Figure 3).

##### Chlorophyll content (90 d).

**Figure 4.**
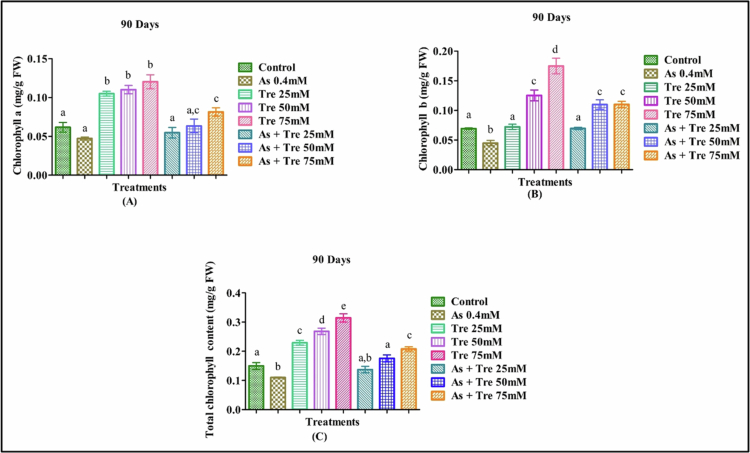
Effect of As and Tre on 90 d old plants of *B. juncea* under various concentrations in comparison with the control plant (A) chlorophyll a, (B) chlorophyll b, and (C) total chlorophyll content. Experimental data represents the mean of triplicates ± standard deviation (SD). Statistical analysis was performed using a one-way ANOVA, with a Bonferroni post-hoc test employed to identify significant differences (*p* < 0.05). Within the figures, distinct superscripts indicate treatments that are significantly different from one another. Treatments included control (without arsenic), and a gradient of arsenic concentration (0.4 mM) and different Tre concentrations (25, 50, and 75 mM).

In 90-d-old *B. juncea* plants, As exposure caused a significant decrease in the content of chlorophyll a, chlorophyll b, and total chlorophyll content by 23.47%, 35.16%, and 35.18% respectively as compared with control. However, the application of trehalose counteracted these reductions in a dose-dependent manner, with the As + Tre 75 mM treatment showing maximum effect where the chlorophyll a, chlorophyll b, and total chlorophyll content increased by 42.02%, 37.11%, and 59.22% respectively over As-treated plants (Figure 4).

Chlorophyll a, the principle photosynthetic pigment in chloroplasts, is integral to the photosynthetic efficiency of plants. As significantly disturbs the chlorophyll level in leaves, making it is a crucial marker of metal toxicity.^34^ The decreased chlorophyll concentration due to As-toxicity can be related to chloroplast ultrastructural changes, the inhibition of chlorophyll-synthesis enzymes, and excess ROS formation, contributing to increased breakdown of pigments and photosynthetic inactivity.^35^ Similar study was conducted by Ahmad et al.^35^ and Praveen et al.^36^ that As reduced the chlorophyll content in *B. juncea*. Another observations were reported by Faisal et al.^19^ As reduced the total chlorophyll content in *B. juncea* plants. Furthermore, As-toxicity damage the synthesis of δ-aminolevulinic acid, a precursor in the chlorophyll biosynthetic pathway and reduce the activity of chlorophyllase and other enzymes involved in chlorophyll production.^37^

Tre plays a vital role in increasing the chlorophyll content under metal toxicity in previous studies. Consistent with previous findings Yang et al.^12^ Tre significantly increased the chlorophyll a and chlorophyll b content in rice plants under Pb toxicity. According to Zulfiqar et al.^38^ Tre significantly increases the chlorophyll a, chlorophyll b, and total chlorophyll content under Cd in *Capsicum annuum* L. Trehalose may protect chloroplast membranes and the photosynthetic machinery by improving antioxidant defense, maintaining membrane stability, and reducing oxidative damage under As-stress conditions. These reports demonstrate that Tre treatment confers protection to membrane integrity and the photosynthetic system against ROS through the decrease of the activity of chlorophyll degradation enzymes under stressful conditions.^39^

#### Carotenoids content

Carotenoids function as important non-enzymatic antioxidants that protect the photosynthetic apparatus from oxidative damage by scavenging ROS and dissipating excess excitation energy.^40^ The decline in carotenoid content under As-stress may therefore be associated with the oxidative degradation of pigments and the disruption of chloroplast metabolism.^41^

**Figure 5.**
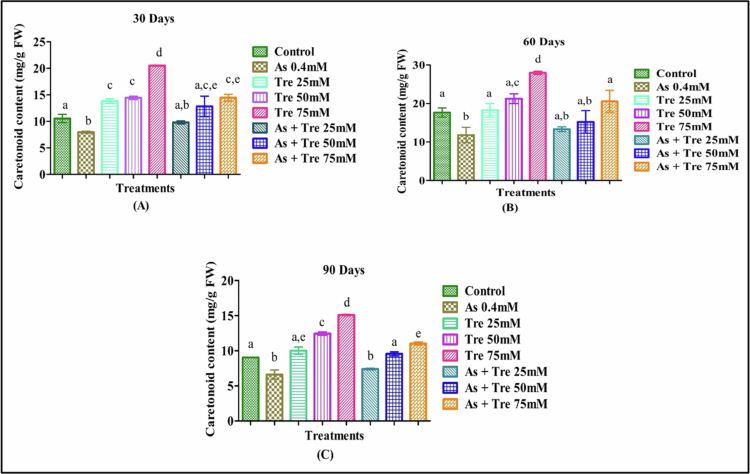
Effect of As and Tre on the carotenoid content of *B. juncea* under various concentrations in comparison with the control plant (A) 30 d, (B) 60 d, and (C) 90 d. Experimental data represents the mean of triplicates ± standard deviation (SD). Statistical analysis was performed using a one-way ANOVA, with a Bonferroni post-hoc test employed to identify significant differences (*p* < 0.05). Within the figures, distinct superscripts indicate treatments that are significantly different from one another. Treatments included control (without arsenic), and a gradient of arsenic concentration (0.4 mM) and different Tre concentrations (25, 50, and 75 mM).

Arsenic stress significantly reduced carotenoid content by 24.48%, 32.96%, and 26.67% in 30, 60, and 90 d old *B. juncea* plants, respectively, compared with those of the control plants. However, trehalose supplementation mitigated the adverse effects of As in a dose-dependent response at all growth stages. Among the treatments, As + Tre 75 mM showed the highest recovery in carotenoid content, with increases of 44.95%, 42.53%, and 40.03% 30-, 60-, and 90-d-old plants, respectively, compared with As-treated plants (Figure 5).

Tre supplementation possibly protects carotenoid biosynthesis and membrane-bound pigments by improving antioxidant defense and maintaining cellular redox homeostasis under stress conditions.^42^

Our findings are consistent with those of previous studies reporting heavy metal-induced depletion of carotenoids. Similar reduction was found in the carotenoid content of *Zea mays* decreases under Cd toxicity, but Tre application increased the carotenoid content.^43^ A similar study also reported that exogenous supplementation of trehalose minimized Cd toxicity and increased the carotenoid content in wheat plants.^44^ In agreement with these findings, the present study showed that Tre effectively alleviated the As-induced reduction in the carotenoid content, indicating its protective role in maintaining photosynthetic pigment stability under stress conditions.

#### Xanthophyll content

Xanthophyll pigments are essential components of the photoprotective system and participate in the xanthophyll cycle, which dissipates excess excitation energy as heat during stress conditions.^45^ Arsenic-induced decline in xanthophyll content may therefore be associated with disruption of chloroplast membranes, oxidative degradation of pigments, and impaired energy dissipation capacity, ultimately affecting photosystem stability.^46^

**Figure 6.**
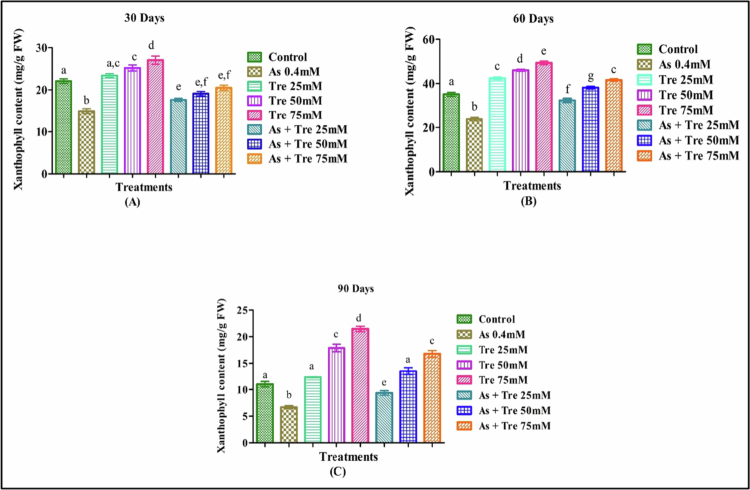
Effect of As and Tre on xanthophyll content of *B. juncea* under various concentrations in comparison with the control plant (A) 30 d, (B) 60 d, and (C) 90 d. Experimental data represents the mean of triplicates ± standard deviation (SD). Statistical analysis was performed using a one-way ANOVA, with a Bonferroni post-hoc test employed to identify significant differences (*p* < 0.05). Within the figures, distinct superscripts indicate treatments that are significantly different from one another. Treatments included a control (without arsenic), and a gradient of arsenic concentration (0.4 mM) and different Tre concentrations (25, 50, and 75 mM).

Arsenic stress significantly reduced xanthophyll 32.17%, 32.40%, and 39.34% at 30, 60, and 90 d old *B. juncea* plants, respectively, compared with control plants. The application of Tre effectively mitigated this reduction in a dose-dependent manner throughout all the growth stages. The greatest restoration was observed under the As + Tre 75 mM treatment, which enhanced xanthophyll content by 27.07%, 42.87%, and 60.23% in 30, 60, and 90-d-old plants, respectively, over the As-stressed plants, suggesting improved protection of photosynthetic pigments against oxidative damage (Figure 6).

Similar reduction has been seen by Majumder et al.^47^ the xanthophyll content is decreased by As in rice plants. The restoration of xanthophyll content following trehalose application suggests improved membrane integrity and enhanced protection of the photosynthetic apparatus through a reduction of ROS-mediated oxidative injury.^48^ Trehalose may also contribute to the stabilization of pigment–protein complexes and maintenance of the cellular redox balance under arsenic stress. According to Ghosh et al.^49^ xanthophyll content of *Triticum aestivum* L. decreased by Cd toxicity. While several studies have investigated the impact of heavy metals stress (As, Cd, and Cr) on overall pigment levels, including carotenoid in various plant species, there is notable lack of research specifically the effect of Tre in modulating xanthophyll accumulation under As stress conditions has not yet been explored *B. juncea*.

#### Anthocyanin content

**Figure 7.**
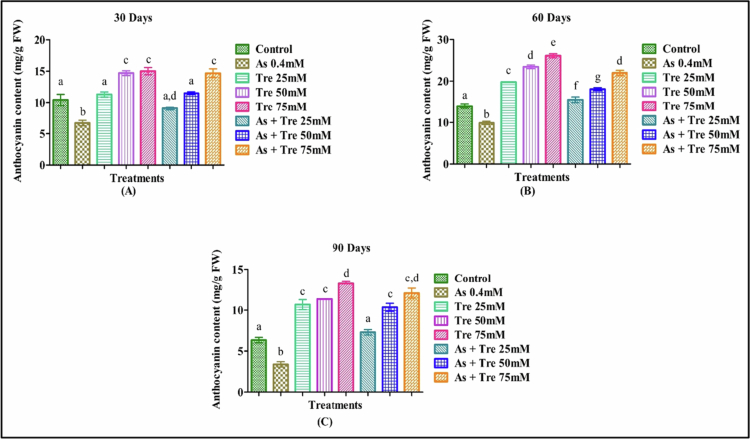
Effect of As and Tre on anthocyanin content of *B. juncea* under various concentrations in comparison with the control plant (A) 30 d, (B) 60 d, and (C) 90 d. Experimental data represents the mean of triplicates ± standard deviation (SD). Statistical analysis was performed using a one-way ANOVA, with a Bonferroni post-hoc test employed to identify significant differences (*p* < 0.05). Within the figures, distinct superscripts indicate treatments that are significantly different from one another. Treatments included a control (without arsenic), and a gradient of arsenic concentration (0.4 mM) and different Tre concentrations (25, 50, and 75 mM).

Anthocyanins are important flavonoid pigments that function as non-enzymatic antioxidants and play a crucial role in protecting plants against As-induced oxidative stress. They efficiently scavenge ROS, reduce lipid peroxidation, stabilize cellular and vacuolar membranes, and protect photosynthetic tissues from oxidative damage.^50^ The reduction in anthocyanin content under metal stress may be attributed to disruption of the phenylpropanoid biosynthetic pathway, oxidative degradation of pigments, and impairment of enzymes involved in anthocyanin synthesis due to excessive ROS accumulation.^51^ Arsenic stress markedly reduced the anthocyanin content by 37.76%, 29.25%, and 40.70% in 30-, 60-, and 90 d old *B. juncea* plants, respectively, relative to the control. In contrast, Tre supplementation markedly improved anthocyanin accumulation across all growth phases in a dose-dependent manner. The strongest response was recorded with the As + Tre 75 mM treatments, which elevated anthocyanin content by 54.40%, 55.02%, and 71.99%, respectively, over that of the As-exposed plants, suggesting enhanced antioxidant protection and improved stress tolerance (Figure 7).

Anthocyanin content due to As stress is reliable with earlier studies. Study conducted by Pandey et al.^52^ the anthocyanin content is decreased by As in *B. juncea* plants. According to Sahay et al.^53^ anthocyanin content of *B. juncea* decreased by As toxicity. Studies have described that anthocyanin plays a vital part in the antioxidant activities and metal detoxification of various plant species.^54^ According to Rehman et al.^55^ Tre application significantly increased the anthocyanin content under Cd toxicity in *Vigna radiata*. Trehalose supplementation probably restored anthocyanin accumulation by maintaining the cellular redox balance, protecting membrane integrity, and stimulating antioxidant and secondary metabolite biosynthetic pathways under stress conditions.^56^ The direct effect of Tre application on the anthocyanin content in many species is not well documented. That is many studies are about Tre biosynthesis and endogenous sugar levels rather than applying Tre externally and measuring anthocyanin changes.

### Oxidative stress markers

#### MDA content

**Figure 8.**
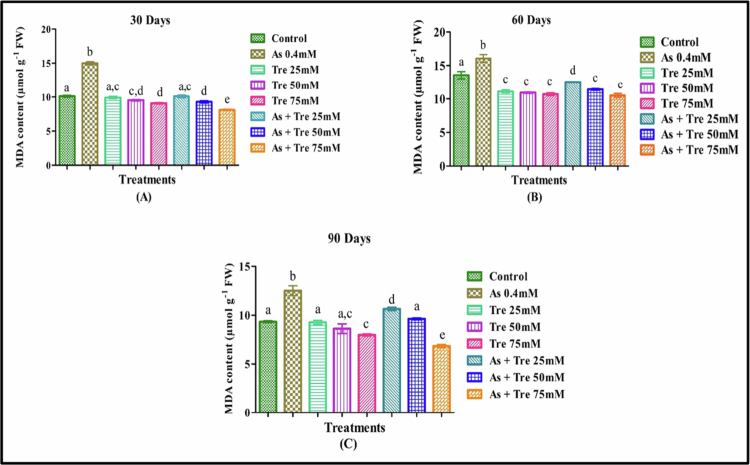
Effect of As and Tre on the MDA activity content of *B. juncea* under various concentrations in comparison with the control plant (A) 30 d (B) 60 d, and (C) 90 d. Experimental data represents the mean of triplicates ± standard deviation (SD). Statistical analysis was performed using a one-way ANOVA, with a Bonferroni post-hoc test employed to identify significant differences (*p* < 0.05). Within the figures, distinct superscripts indicate treatments that are significantly different from one another. Treatments included a control (without arsenic), and a gradient of arsenic concentration (0.4 mM) and different Tre concentrations (25, 50, and 75 mM).

Arsenic treatment caused a pronounced increase in the MDA content by 32.57%, 15.53%, and 25.42% in 30, 60, and 90 d old *B. juncea* plants, respectively, compared with untreated plants, indicating severe oxidative injury. However, exogenous Tre markedly suppressed lipid peroxidation across all growth stages. Among the treatments, As + Tre 75 mM exhibited the most substantial effect, lowering the MDA content by 45.93%, 25.50%, and 49.60%, respectively, relative to As-stressed plants, thereby demonstrating enhanced cellular protection under stress conditions (Figure 8).

Similar findings were reported by Raza et al.^57^ under Pb toxicity *B. juncea* plants showed a large increase in the MDA content. The marked reduction in the MDA content following trehalose supplementation suggests that trehalose alleviates As-induced oxidative membrane injury by strengthening antioxidant defense systems and stabilizing membrane structures. Trehalose may protect membrane lipids and proteins through osmoprotective action, maintenance of cellular hydration, preservation of membrane integrity, and enhancement of enzymatic and non-enzymatic antioxidant activities that collectively suppress ROS-mediated peroxidative damage under As stress conditions.^58^ Faisal et al.^19^ As increased the concentration of MDA in *B. juncea*. Reduced MDA levels indicates that Tre stabilizes membranes and enhances basal antioxidant capacity. Earlier findings suggested that under normal conditions trehalose reduces the MDA content in *B*. *napus* L. The decrease in MDA was due to the decline in membrane impairment.

#### H_2_O_2_ content

Arsenic disrupts electron transport processes in chloroplasts and mitochondria, causing leakage of electrons toward molecular oxygen and thereby generating superoxide radicals (O₂•^−^). These radicals are subsequently converted into H₂O₂ by superoxide dismutase (SOD).^59^ Under prolonged As exposure, overproduction of H₂O₂ exceeds the detoxification capacity of antioxidant enzymes such as CAT and APX, leading to oxidative damage to membrane lipids, proteins, nucleic acids, and photosynthetic components.^60^ Elevated H₂O₂ levels also enhance membrane permeability and impair cellular metabolism through disruption of redox-sensitive pathways.^61^

**Figure 9.**
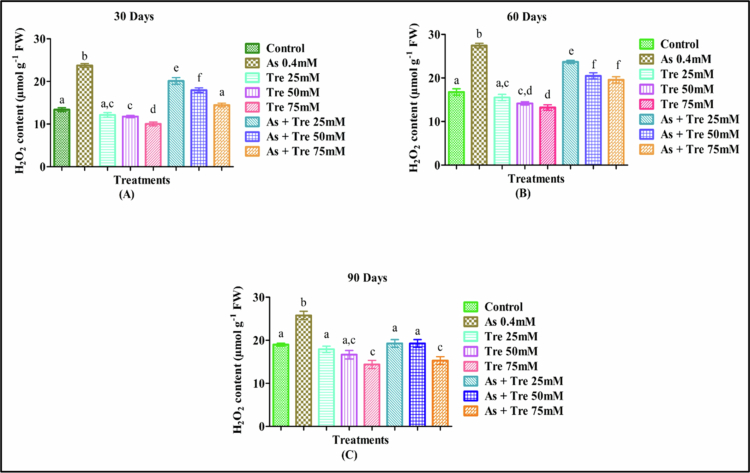
Effect of As and Tre on H_2_O_2_ content of *B. juncea* under various concentrations in comparison with the control plant (A) 30 d, (B) 60 d, and (C) 90 d. Experimental data represents the mean of triplicates ± standard deviation (SD). Statistical analysis was performed using a one-way ANOVA, with a Bonferroni post-hoc test employed to identify significant differences (*p* < 0.05). Within the figures, distinct superscripts indicate treatments that are significantly different from one another. Treatments included control (without arsenic), and a gradient of arsenic concentration (0.4 mM) and different Tre concentrations (25, 50, and 75 mM).

Increased H_2_O_2_ accumulation was observed in As-treated *B. juncea* plants by 43.65%, 38.54%, and 26.35% at 30, 60, and 90 d old *B. juncea* plants, respectively, compared with the control. The application of Tre effectively minimized ROS accumulation at all developmental stages. Among the treatments, As + Tre 75 mM exhibited the strongest response, decreasing the H_2_O_2_ content by 39.13%, 47.31%, and 40.67% in the plants relative to the As-stressed plants, indicating improved oxidative balance and stress tolerance (Figure 9).

Similar observations were observed by Bhardwaj et al.^62^ that H_2_O_2_ levels were increased under As-stress in *Raphanus sativus*. As perturbs photosynthetic electron flow (chloroplast) and mitochondrial respiration; leaked electrons reduce O₂ to superoxide (O₂•^−^), which is quickly converted to H₂O₂ by SOD.^61^

The marked reduction in H₂O₂ accumulation following trehalose supplementation suggests that trehalose improves cellular redox homeostasis by enhancing antioxidant defense systems, stabilizing membrane structures, protecting organellar integrity, and limiting ROS overproduction under As-stress conditions. Therefore, Tre plays a significant role in increasing the level of H₂O₂ under metal stress. Similar study was conducted by Rehman et al.^55^ they concluded that under metal toxicity like Cd increased the H_2_O_2_ content under Tre treatment in *Vigna radiata*.

#### Enzymatic antioxidants

#### Catalase activity

**Figure 10.**
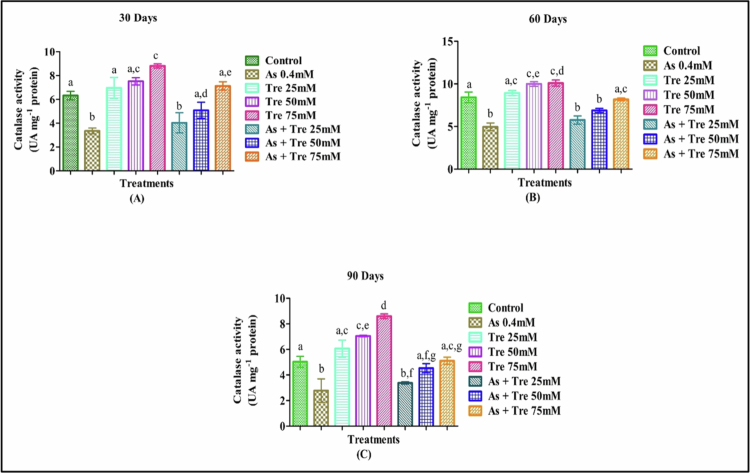
Effect of As and Tre on CAT activity content of *B. juncea* under various concentrations in comparison with the control plant (A) 30 d, (B) 60 d, and (C) 90 d. Experimental data represents the mean of triplicates ± standard deviation (SD). Statistical analysis was performed using a one-way ANOVA, with a Bonferroni post-hoc test employed to identify significant differences (*p* < 0.05). Within the figures, distinct superscripts indicate treatments that are significantly different from one another. Treatments included control (without arsenic), and a gradient of arsenic concentration (0.4 mM) and different Tre concentrations (25, 50, and 75 mM).

Catalase (CAT) is a major antioxidant enzyme responsible for the decomposition of H₂O₂ into water and oxygen during oxidative stress. As-induced reduction in CAT activity may result from excessive ROS accumulation, oxidative inactivation of enzyme proteins, and disruption of the cellular redox balance.^63^ Arsenic stress caused a substantial decline in CAT activity by 46.95%, 41.08%, and 44.71% at 30, 60, and 90 d old *B. juncea* plants, respectively, compared with those of the control plants, indicating impairment of the antioxidant defense system. However, Tre application improved the CAT activity across all the growth stages. The maximum recovery was observed under the As + Tre 75 mM treatment, which enhanced CAT activity by 52.84%, 39.13%, and 45.69%, respectively, compared with that of the control plants, suggesting improved ROS detoxification and oxidative stress tolerance (Figure 10).

The reduction in CAT activity observed under As toxicity in the present study is consistent with previous reports. According to Kanwar et al.^64^ As reduced the CAT content in *B. juncea*. Additionally, Ahmad et al.^35^ they concluded that As reduced CAT activity in *B. juncea* plants. Restoration of CAT activity following trehalose treatment suggested enhanced ROS detoxification, stabilization of antioxidant enzymes, and improved maintenance of cellular oxidative homeostasis under As-stress conditions. Previous studies have concluded that Tre plays a significant role in increasing CAT activity under heavy metals stress. Yang et al.^12^ demonstrated that Tre increasing the CAT activity in *Oryza sativa* under Pb toxicity. Comparable study were conducted by Yan et al.^65^ that under As stress decreases CAT activity but Tre increasing CAT activity in *Oryza sativa*.

#### Ascorbate peroxidase activity

**Figure 11.**
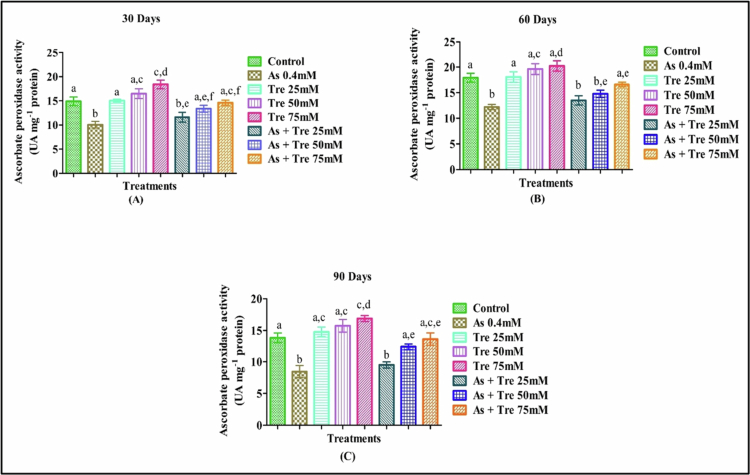
Effect of As and Tre on APX activity content of *B. juncea* under various concentrations in comparison with the control plant (A) 30 d, (B) 60 d, and (C) 90 d. Experimental data represents the mean of triplicates ± standard deviation (SD). Statistical analysis was performed using a one-way ANOVA, with a Bonferroni post-hoc test employed to identify significant differences (*p* < 0.05). Within the figures, distinct superscripts indicate treatments that are significantly different from one another. Treatments included control (without arsenic), and a gradient of arsenic concentration (0.4 mM) and different Tre concentrations (25, 50, and 75 mM).

Ascorbate peroxidase (APX) is a crucial antioxidant enzyme of the ascorbate–glutathione cycle that detoxifies H₂O₂ by converting it into water using ascorbate as an electron donor, particularly within chloroplasts, where CAT activity is limited.^6^ The decline in APX activity was recorded as 32.47%, 31.87%, and 38.96% at 30, 60, and 90 d old *B. juncea* plants compared with that of the control plants. Reduced APX activity may further enhance H₂O₂ accumulation, resulting in oxidative damage to chloroplast membranes, the photosynthetic machinery, proteins, and nucleic acids.^66^ In contrast, the application of Tre effectively increased APX activity during various growth stages in a dose-dependent pattern. The highest stimulation was observed under As + Tre 75 mM treatment, which increased APX activity by 31.15%, 26%, and 38.10% respectively, suggesting improved ROS detoxification and cellular protection (Figure 11).

Bhardwaj et al.^67^ As (0.7 mM), reported that As decreased the APX activity in *Raphanus sativus* which is also belongs to the *Brassicaceae* family as compared with control plants. Study conducted by Hasanuzzaman et al.^68^ they concluded that the maximum decline in the activities of the APX was observed in *Oryza sativa* under Cd stress. The enhancement of APX activity following trehalose supplementation suggests improved regulation of the ascorbate–glutathione cycle and more efficient ROS detoxification under stress conditions.^69^ Trehalose may protect APX and other antioxidant enzymes from oxidative denaturation by stabilizing membrane structures, maintaining cellular hydration, preserving protein conformation, and sustaining intracellular redox homeostasis, thereby enhancing oxidative stress tolerance under stress conditions.^70^ Previous study further demonstrated that Tre increased APX activity in *Zea mays* under Cr stress.^43^

#### Superoxide dismutase activity

SOD activity decreased significantly in 30 d old of *B. juncea* plants by 34.65% due to As toxicity in comparison to control plants. Tre 25 mM, Tre 50 mM, and Tre 75 mM showed a significant enhancement by 10.70%, 19.16%, and 26.14% by Tre treatment. In comparison to As plants samples along with Tre possessed increase in SOD activity by 15.19%, 23.01%, and 31.89% in As + Tre 25 mM, As + Tre 50 mM, and As + Tre 75 mM respectively as compared with As stressed alone plants.

**Figure 12.**
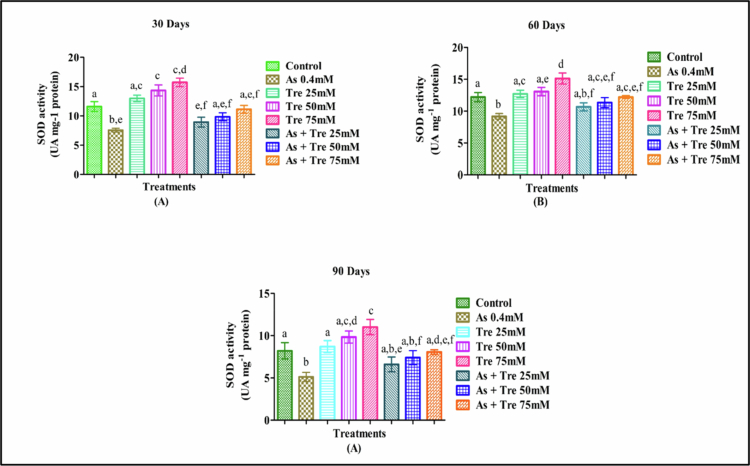
Effect of As and Tre on SOD activity content of *B. juncea* under various concentrations in comparison with the control plant (A) 30 d, (B) 60 d, and (C) 90 d. Experimental data represents the mean of triplicates ± standard deviation (SD). Statistical analysis was performed using a one-way ANOVA, with a Bonferroni post-hoc test employed to identify significant differences (*p* < 0.05). Within the figures, distinct superscripts indicate treatments that are significantly different from one another. Treatments included a control (without arsenic), and a gradient of arsenic concentration (0.4 mM) and different Tre concentrations (25, 50, and 75 mM).

Superoxide dismutase (SOD), serves as the first line of antioxidant defense by catalyzing the dismutation of superoxide radicals (O₂•^−^) into H₂O₂ and molecular oxygen, thereby preventing excessive oxidative damage within plant cells.^71^ Arsenic exposure significantly suppressed SOD activity in *B. juncea* plants, causing reduction of 34.65%, 24.80%, and 37.68% at 30, 60, and 90 days respectively compared with those of the control plants. Under As-stress, disruption of the chloroplast and mitochondrial electron transport systems enhances superoxide radical generation, while excessive ROS accumulation may oxidatively inactivate SOD proteins and disturb cellular redox homeostasis, leading to reduced enzyme activity.^72^ Suppression of SOD activity consequently promotes oxidative injury to membranes, proteins, nucleic acids, and the photosynthetic machinery.^73^ In contrast, treatment with Tre enhanced SOD activity at all growth stages, with the maximum stimulation observed under the As + Tre 75 mM combination. This treatment increased SOD activity by 31.89%, 24.82%, and 36.59%, respectively, relative to As-treated plants, indicating improved enzymatic defense against ROS-induced oxidative damage (Figure 12).

Similar reduction in SOD activity under As toxicity have been reported in previous studies. This decline may be attributed to the degradation of proteins, impairment of nucleic acids, and enzyme inhibition induced by ROS, which were exacerbated by the prolonged treatment period.^72^ Earlier findings suggested that As decreased the SOD activity in *Oryza sativa* Bhadwal et al.^74^ The restoration of SOD activity following trehalose supplementation suggests improved regulation of ROS metabolism and stabilization of antioxidant enzymes under stress conditions. Trehalose maybe protects SOD activity by preserving protein conformation, maintaining membrane integrity, reducing oxidative denaturation, and enhancing the cellular redox balance, thereby strengthening enzymatic antioxidant defense against As-induced oxidative stress. Previous studies have concluded that,^12^ Tre increased SOD activity in *Oryza sativa* under Pb toxicity.^12^ Similarly, Hasanuzzaman et al.^66^ they concluded that Tre application SOD activity increased respectively, in As-stressed seedlings of relative to control *Oryza sativa*.

#### Glutathione reductase activity

**Figure 13.**
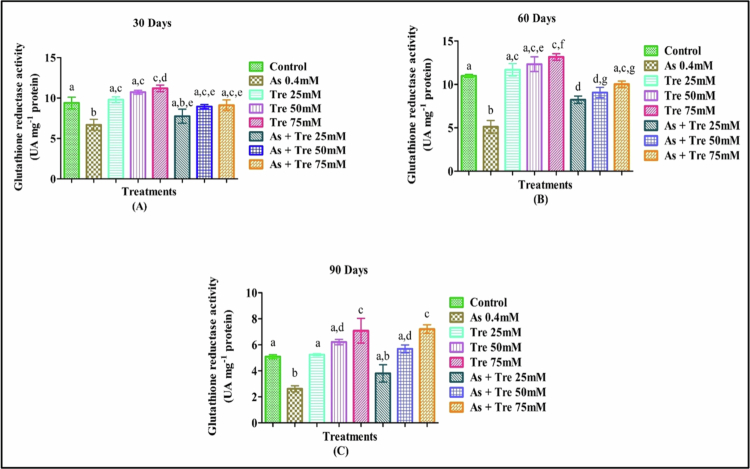
Effect of As and Tre on GR activity content of *B. juncea* under various concentrations in comparison with the control plant (A) 30 d, (B) 60 d, and (C) 90 d. Experimental data represents the mean of triplicates ± standard deviation (SD). Statistical analysis was performed using a one-way ANOVA, with a Bonferroni post-hoc test employed to identify significant differences (*p* < 0.05). Within the figures, distinct superscripts indicate treatments that are significantly different from one another. Treatments included a control (without arsenic), and a gradient of arsenic concentration (0.4 mM) and different Tre concentrations (25, 50, and 75 mM).

Glutathione reductase (GR) is a crucial component of the ascorbate–glutathione cycle that regenerates reduced glutathione (GSH) from its oxidized form (GSSG), thereby maintaining intracellular redox homeostasis during oxidative stress.^75^ GR activity decreased by 28.65%, 53.57%, and 48.44% in 30-, 60-, and 90-d-old plants, respectively, compared with that in control plants. Under As-toxicity, excessive ROS accumulation may disrupt glutathione metabolism, oxidatively damage enzyme proteins, and deplete cellular reducing power, resulting in the suppression of GR activity.^63^ Reduced GR activity limits GSH regeneration, weakens ROS detoxification capacity, and enhances oxidative injury to membranes, proteins, and nucleic acids.^76^ Tre supplementation markedly improved GR activity throughout different growth stage in a dose-dependent manner. The maximum enhancement was observed under As + Tre 75 mM treatment, which increased the GR activity by 26.58%, 48.98%, and 63% compared with As-stressed plants, suggesting improved glutathione regeneration and increased antioxidant defense under As-toxicity (Figure 13).

A study conducted by Popov et al.^77^ they concluded that As reduced the GR activity in *Oryza sativa* as compared with that in the control plants. Similarly according to Algopishi et al.^78^ As decreased the activity of GR in *B. napus* plants. Another study conducted by Yan et al.^65^ Tre increased APX activity compared with that of control plants. The enhancement of GR activity following trehalose supplementation suggests improved maintenance of the glutathione pool and more efficient regulation of the cellular redox balance under stress conditions.^43^ Trehalose may stabilizes antioxidant enzymes and membrane structures, preserves cellular hydration, and protects thiol-containing proteins from oxidative denaturation, thereby improving glutathione-dependent antioxidant defense against As-induced oxidative stress.

### Non-enzymatic antioxidants

#### Flavonoid content

**Figure 14.**
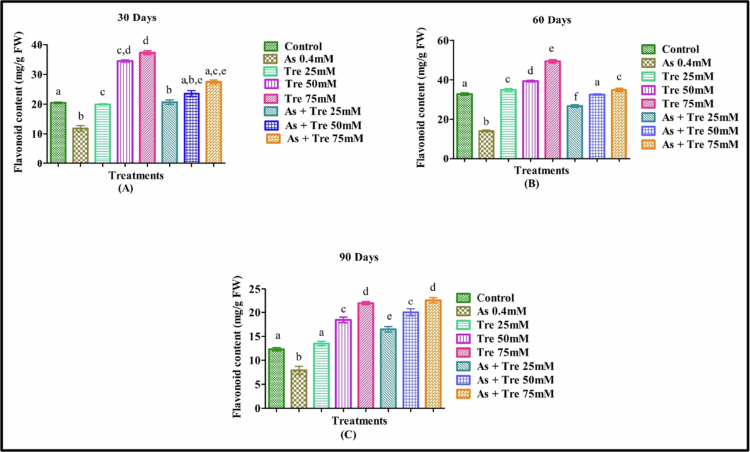
Effect of As and Tre on the flavonoid content of *B. juncea* under various concentrations in comparison with the control plant (A) 30 d, (B) 60 d, and (C) 90 d. Experimental data represents the mean of triplicates ± standard deviation (SD). Statistical analysis was performed using a one-way ANOVA, with a Bonferroni post-hoc test employed to identify significant differences (*p* < 0.05). Within the figures, distinct superscripts indicate treatments that are significantly different from one another. Treatments included a control (without arsenic), and a gradient of arsenic concentration (0.4 mM) and different Tre concentrations (25, 50, and 75 mM).

Flavonoids are important secondary metabolites that function as non-enzymatic antioxidants under heavy metal stress conditions. They protect plant cells by scavenging ROS, chelating toxic metal ions, stabilizing cellular membranes, and preventing oxidative damage to proteins, lipids, and nucleic acids.^40^ Arsenic toxicity significantly altered the flavonoid content in *B. juncea* plants at different growth phases. The flavonoid content increased by 37.35% and 26.82% in the 30- and 60-d old plants, whereas a slight reduction of 3.98% was observed at 90 d compared with that in the control plants. The increased flavonoid accumulation observed under As-stress may therefore represent an adaptive defense response against excessive ROS generation and metal-induced oxidative injury.^79^ Trehalose application further increased flavonoid accumulation throughout plant growth in a concentration-dependent manner. The highest response was recorded under the As + Tre 75 mM treatment, which elevated flavonoid content by 41.87%, 33.34% and 48.06% in all the growth stages as compared with As-stressed plants, indicating improved non-enzymatic antioxidant protection (Figure 14).

The ability of plant flavonoids to form stable chelates with heavy metal ions serves as a key defensive mechanism, mitigating the deleterious effects of metal stress on plant physiology.^80^ It has been reported that As reduced the flavonoid content in the leaves of spinach as compared with that in control plants.^77^ Similarly, Bhardwaj et al.^68^ flavonoid content drastically declined in As-stressed *Raphanus sativus* plants. Flavonoids are synthesized through the phenylpropanoid pathway. Tre treatment has been shown to up-regulate key enzymes such as phenylalanine ammonia-lyase (PAL) and chalcone synthase (CHS), leading to greater flux into flavonoid biosynthesis.^81^ According to Algopishi et al.^78^ Tre application resulted in increase of the flavonoids accumulation in *Trigonella foenum-gracum* plants. Similarly, Tre increased the total flavonoid content in *Vigna radiata* plants under Zn toxicity.^82^

#### Protein content

**Figure 15.**
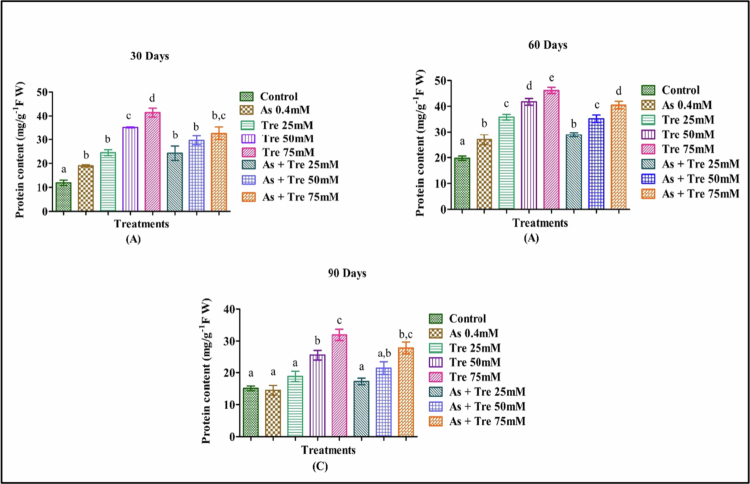
Effect of As and Tre on the protein content of *B. juncea* under various concentrations in comparison with the control plant (A) 30 d, (B) 60 d, and (C) 90 d. Experimental data represents the mean of triplicates ± standard deviation (SD). Statistical analysis was performed using a one-way ANOVA, with a Bonferroni post-hoc test employed to identify significant differences (*p* < 0.05). Within the figures, distinct superscripts indicate treatments that are significantly different from one another. Treatments included a control (without arsenic), and a gradient of arsenic concentration (0.4 mM) and different Tre concentrations (25, 50, and 75 mM).

Arsenic stress significantly affected the level of protein synthesis in *B. juncea* plants at different stages. There was an increase in protein content of 37.36% and 26.80% for 30- and 60-d-old plants, while there was a small decrease in protein content of 4.15% in 90-d-old plants compared with the controls. Alterations in protein accumulation under As-stress may represent a complex cellular response involving both stress-induced synthesis of protective proteins and oxidative degradation of existing proteins.^83^ Excessive ROS accumulation further promotes oxidative modification and fragmentation of proteins, ultimately affecting protein stability and turnover under prolonged stress conditions.^72^ The addition of trehalose also contributed to an increase in protein content depending on its concentration. Among all the treatments, As + Tre 75 mM treatment resulted in the highest increase in protein content of 41.86%, 33.32%, and 48.06% in the 30-, 60-, and 90-d-old plants, respectively, compared to the stressed plants) (Figure 15).

Previous studies have reported alterations in protein content in *B. juncea* under As toxicity. Comparable declines were observed by Kanwar et al.^64^ protein content shows reduction under As toxicity in *B. juncea* plants. Ali et al.^84^ they concluded that As led to a significantly higher increase in protein content in *B. juncea* plants. Arsenic toxicity disrupts protein structure and enzyme activity through interaction with thiol groups, leading to oxidative damage and cellular dysfunction. Trehalose serves as both an energy source and a protective osmolyte, stabilizing proteins and membranes against dehydration-induced damage. Its unique chemical properties allow it to preserve cellular structural integrity and mitigate oxidative impairment.^38^ Rehman et al.^55^ they concluded that Tre increases the protein content in *V. radiata* under Cd stress.

#### Glycine betaine content

**Figure 16.**
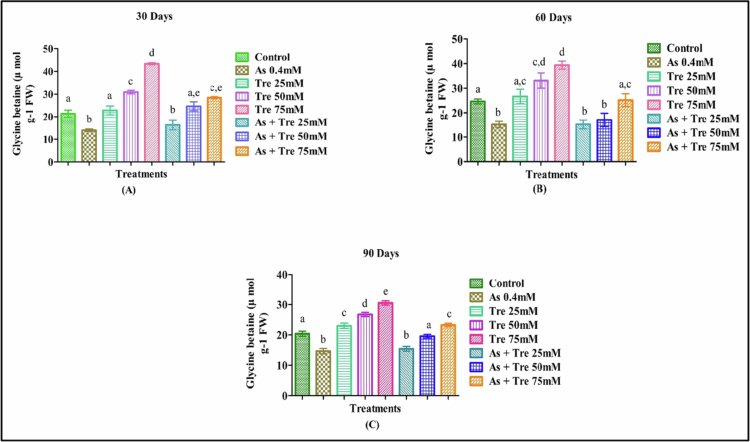
Effect of As and Tre on GB content of *B. juncea* under various concentrations in comparison with the control plant (A) 30 d, (B) 60 d, and (C) 90 d. Experimental data represents the mean of triplicates ± standard deviation (SD). Statistical analysis was performed using a one-way ANOVA, with a Bonferroni post-hoc test employed to identify significant differences (*p* < 0.05). Within the figures, distinct superscripts indicate treatments that are significantly different from one another. Treatments included a control (without arsenic), and a gradient of arsenic concentration (0.4 mM) and different Tre concentrations (25, 50, and 75 mM).

As a primary osmoprotectant, GB is essential for plant stress tolerance. Its accumulation helps stabilize metabolism and support development under unfavorable conditions. These benefits are attained through several combined mechanisms, including the preservation of redox balance and the prevention of oxidative damage by free radicals.^81^ Arsenic stress showed a strong impact on reducing the GB content, showing a decrease of 34.66%, 38.63%, and 28.01% in 30-, 60-, and 90-d-old *B. juncea* plants, respectively, as compared with control plants. The addition of Tre showed an significant enhancement in elevating GB content at all growth stages in a dose-dependent manner. As + Tre 75 mM showed a remarkable effect on the increase in GB content by 50.97%, 39.92%, and 36.93% in 30-, 60-, and 90-d-old plants, respectively (Figure 16).

According to Bhardwaj et al.^62^ As stress inhibited the GB content in *Raphanus sativus* plants. Although GB typically accumulates under abiotic stress, some studies have reported its decline under extreme conditions, e.g. heat stress in *Q. robur*.^85^ This suggests that intense stress can impair GB synthesis or accelerate its degradation.

Tre and GB are both acts as a compatible solute, Tre increased the GB content under drought stressed in *Ocimum basilicum* plants.^38^ There is very limited information about the natural GB dynamics in plants exposed to heavy metals. Most current studies focus on exogenous GB supplementation and its protective roles rather than quantifying how endogenous GB levels changes in response to increasing metal concentrations and exposure durations.

Trehalose possibly stabilizes membrane structures and proteins, preserves cellular hydration, maintains ionic and redox homeostasis, and supports metabolic pathways associated with compatible solute accumulation, thereby improving cellular tolerance against As-induced oxidative and osmotic stress.

The findings of the present study also have broader agricultural and environmental significance. Arsenic contamination is an increasing threat to crop productivity, food safety, and soil health in many agricultural regions worldwide. The observed protective role of trehalose against As-induced oxidative damage suggests its potential application as an eco-friendly and sustainable stress ameliorant for improving crop performance under heavy metal stress conditions. Moreover, the ability of *B. juncea* to tolerate As-stress through enhanced antioxidant and osmoprotective mechanisms may contribute to its potential use in phytoremediation programs for As-contaminated soils. Therefore, trehalose-mediated stress regulation may provide a promising strategy for sustainable agriculture and environmental management in metal-polluted ecosystems.

## Conclusion

As shown by the current research, As-toxicity affects the physiological and biochemical status of *B. juncea* through oxidative stress, damage to photosynthetic pigments, inhibition of the antioxidant system, and disruption of the osmotic state. The excessive production of ROS caused lipid peroxidation in the membranes of plant cells and resulted in their oxidative stress and damage, causing a decline in the efficiency of enzymatic and non-enzymatic antioxidants.

The application of trehalose externally provided a significant mitigation against As-induced phytotoxicity in a dose-dependent manner and improved the ability of plants to tolerate stress at various growth stages. The protective action of trehalose was linked to the stabilization of photosynthetic pigments, lower levels of oxidative stress biomarkers, increased activity of antioxidant enzymes, and higher amounts of antioxidants, including flavonoids, proteins, and glycine betaine. The beneficial effect of trehalose could possibly be due to its role in ROS removal, maintenance of cell membrane stability, cellular water content, osmoregulation, and stabilization of antioxidant systems under stress conditions. It can also be seen that the enhanced regulation of the ascorbate-glutathione cycle is another major advantage of using trehalose.

In addition to being a component of osmoprotection, trehalose is also involved in stress signaling pathways through the regulation of antioxidant systems that are responsive to ROS, redox-controlled metabolism, and stress-related gene expression. All the treatments were compared in terms of their effectiveness, and it was found that 75 mM trehalose showed the maximum protective effect, which suggests better efficacy of this substance in triggering the processes of defense and detoxification of the As-induced oxidative stress. In general, the results of this investigation suggest that trehalose is an excellent osmoprotectant that may serve as an environmentally friendly strategy to enhance As-tolerance of *B. juncea*.

## Future recommendations

Future studies should focus on more research at the molecular and transcriptional levels aimed at understanding the regulation of genes that respond to ROS and antioxidant signaling as well as As toxicity response mechanisms through trehalose biosynthesis in response to As. Additionally, extensive field-level validation trials would be required to assess the practical applicability of trehalose under natural agricultural conditions. Studies on As-uptake, accumulation, transport, and translocation in plants may provide further insights into the function of trehalose in limiting arsenic absorption.

## Data Availability

Data will be made available on request.
